# Prosocial perceptions of taxation predict support for taxes

**DOI:** 10.1371/journal.pone.0225730

**Published:** 2019-11-26

**Authors:** Emily M. Thornton, Lara B. Aknin, Nyla R. Branscombe, John F. Helliwell

**Affiliations:** 1 Department of Psychology, Simon Fraser University, Burnaby, British Columbia, Canada; 2 Department of Psychology, University of Kansas, Lawrence, Kansas, United States of America; 3 Vancouver School of Economics, University of British Columbia, Vancouver, British Columbia, Canada; University of Brescia, ITALY

## Abstract

Many people report disliking taxes despite the fact that tax funds are used to provide essential services for the taxpayer and fellow citizens. In light of past research demonstrating that people are more likely to engage in prosocial action when they recognize how their assistance positively impacts the recipient, we examine whether recognition of how one’s tax contributions help other citizens–*perceived prosocial taxation*–predicts more supportive views of taxation and greater engagement. We conducted three correlational studies using North American samples (*N* = 902, including a nationally representative sample of over 500 US residents) in which we find that perceived prosocial taxation is associated with greater enjoyment paying taxes, willingness to continue paying taxes, and larger financial contributions in a tax-like payment. Findings hold when controlling for several demographic variables, participants’ general prosocial orientation, and the perception that tax dollars are being put to good use. In addition, we examined data from six waves of the World Values Survey (*N* > 474,000 across 107 countries). We find that people expressing trust in their government and civil service–thereby indicating some confidence that their taxes will be used in prosocial ways–are significantly more likely to state that it is never justifiable to cheat on taxes. Together, these studies offer a new and optimistic perspective on taxation; people may hold more positive views and be more willing to contribute if they believe their contribution benefits others.

## Introduction

Most nations collect taxes from citizens to support essential public services, such as education, health, infrastructure, and policing. Yet, many people express deep discontent about paying their taxes. For instance, in the United States, nearly 20% of Americans identify with the message of the Taxed Enough Already Party [1] and research suggests that some people will go to extreme lengths to avoid paying taxes, even in comparison to equal monetary costs [2]. How can taxation be seen in a more favourable light? We propose that people who recognize how their tax dollars will be used to help others are more likely to see taxation positively and express a greater willingness to contribute. We examined this possibility in a series of four studies.

Governments around the world collect hundreds of millions in tax revenue annually [3]. A substantial portion of these tax dollars are intended to support programs that benefit citizens, such as public schools, roads, health care, public housing, police, the military and more. Although nearly every citizen benefits from some of these services [4], dislike for taxation is surprisingly common. Indeed, while the amount of revenue lost from tax avoidance or aversion is difficult to measure, it is estimated to be large in all countries, whether from the shadow economy and non-filing [5] or from illicit international transfers [6]. Therefore, there is much to be gained from identifying factors that increase voluntary tax compliance.

Prosociality may be one such factor. Prosociality is the tendency to engage in kind or generous behaviour for the benefit of others (e.g., [7]), even when costly for oneself. Displayed in various forms, such as spending time volunteering, donating blood, and using one’s financial resources to buy gifts for others or make charitable donations, prosocial action is observed and valued around the world [8], suggesting that it may represent a fundamental feature of human behaviour [9]. Indeed, prosociality is not only ubiquitous, but past research demonstrates that using one’s financial resources to help others promotes well-being in rich and poor countries around the globe [10–11]. Thus, given that taxation redirects personal income toward public goods shared by fellow citizens, people who recognize the prosocial nature of taxes may find taxes less aversive or even worthwhile.

Past research supports the notion that broad prosocial attitudes are associated with positive views of taxation. Indeed, Brizi and colleagues [12] found that prosocial inclinations are associated with increased tax compliance in two correlational studies. Moreover, when participants were primed with a prosocial value orientation (vs. proself orientation), they reported higher intentions to pay taxes [12]. In other words, participants who were primed to prioritize the public good over their personal goals were more supportive of taxation. Other research has found that seeing fellow citizens in a positive light is correlated with favorable tax perceptions [13]. Finally, experimental evidence indicates that people reminded that wealth entails a responsibility to help others viewed taxation more positively than people who read neutral statements [14]. Together, these findings suggest that a prosocial orientation–or general tendency to care about the needs of others–is associated with and leads to greater support for taxation. But how might an individual’s perception of a tax in and of itself predict support for taxation, above and beyond a general prosocial orientation? As far as we are aware, no research has investigated this question.

While a broad prosocial orientation or obligation to others may lead to more favorable impressions of taxes, the present research investigates whether people who recognize the specific positive impact taxation can have on others, which we refer to as *perceived prosocial taxation*, are more supportive of taxation and willing to pay taxes. In particular, we propose that people who recognize the ways in which their money is being used to help others are more likely to see the value of taxation, report a greater willingness to contribute, and provide more money in tax-like transfers. This hypothesis is grounded in past research demonstrating that *prosocial impact*–information about how one’s actions or behavior positively influence others–is a key motivator of generosity [15–16]. For instance, in one longitudinal quasi-experiment, university fundraising callers either met with a student who benefited from their efforts or did not. Fundraising callers who met with a beneficiary exhibited greater commitment to their work, in that these callers went on to spend more time on the phone with donors and amassed larger donations [15]. Indeed, callers who were able to see the positive impact that their assistance was having on others showed increased motivation to continue fundraising. Thus, past work demonstrates how awareness of prosocial impact can encourage and sustain charitable action. Here, we extend the idea of prosocial impact to a new domain: taxation. Specifically, we offer the first examination of whether recognizing the benefit that one’s tax dollars have on others predicts positive perceptions and support for taxes in a series of studies, including a nationally representative sample of Americans and large international survey. The latter sample also helps to show that tax compliance is higher where respondents have greater confidence that their governments are more willing and able to use tax revenues effectively to deliver public services.

### Present research

We report four correlational studies in which we investigate whether people who recognize the ways that taxation benefits others are more willing to support taxation, report enjoyment from it, and contribute money to it. In Study 1 we asked a nationally representative panel of over 500 Americans to report the extent to which they felt their taxes benefited others in society, their view of taxes, and their willingness to pay taxes. In Study 2 we conducted a lab study in which students earned a $5 study payment which was then taxed at 20%. After the tax was collected, students rated the extent to which they thought their tax dollars would have a beneficial impact on other students, to what extent they enjoyed paying the tax, to what extent the tax was worthwhile, and to what extent they thought the tax should continue. In Study 3, another group of students earned up to $5 and were told that they could contribute some of their earnings to a student tax supporting an on-campus student society. Participants reported to what extent they thought their tax dollars would help other students, their view of taxes, their support for the continuation of the lab tax, and how much money they would provide to the student society. We examined whether feelings of perceived prosocial taxation predicted greater tax endorsement (Studies 1–3), enjoyment (Studies 1–3), and contribution (Study 3). Finally, in Study 4, we analyzed data from the World Values Survey (6 waves, *N* > 474,000) to investigate whether respondents with a great deal of confidence in their governments and civil services–thereby indicating optimism that their taxes will be used in prosocial ways–are significantly more likely to state that it is never justifiable to cheat on taxes.

Participant written consent was obtained in Studies 1–3, and procedures were approved by the Simon Fraser University Research Ethics Board (2014s0136 and 2017s0082). Data analyzed in Study 4 are exempt from ethical review because they are publicly available (see TCPS ethics, Article 2.2).

## Study 1

### Material and methods

#### Participants

In March 2016, a national sample of 501 Americans (*M*_*age*_ = 48.53, *SD* = 15.83, 50.1% male, 49.9% female) was recruited through Qualtric’s survey panel. The sample matched the US distributions for age, gender, and income, and was intentionally run in the weeks leading up to the US federal income tax deadline (April 15^th^) because we expected most Americans to be completing their taxes at that time.

#### Procedure

As part of a larger study, participants indicated to what extent they agreed with the statements “I am aware of the ways in which paying taxes is benefiting my society” and “I feel that I can have a positive impact on my society through paying taxes” from 1 (Strongly disagree) to 7 (Strongly agree). These items were highly correlated, *r*(499) = 0.75, *p <* 0.001, so scores were averaged to create a single measure of perceived prosocial taxation.

Participants also completed several questions assessing their support for taxation. Specifically, participants responded to “How positively or negatively do you view your taxes?” from 0 (Negative) to 100 (Positive), “To what extent are you willing to pay your taxes?” from 0 (Not at all) to 100 (Extremely), and finally, “How satisfied are you paying your taxes?” from 0 (Not at all) to 100 (Extremely). To control for general prosociality, participants reported their general prosocial tendencies by rating their agreement with “In general, I am the kind of person who enjoys helping other people” on a 1 (Strongly disagree) to 7 (Strongly agree) scale. Finally, participants provided demographic information. In particular, participants reported their household income (1 = Under $15,000 to 7 = Over $100,000), as well as their age, gender (1 = Male, 2 = Female, 3 = Other, 4 = Prefer not to say), and political orientation (1 = Very conservative to 7 = Very liberal).

All studies included a manipulation in which participants were randomly assigned to think about taxes as being self-serving or other-serving. This manipulation did not impact the outcome measures in any study. As such, participants were collapsed across conditions for analyses.

### Results

We examined whether perceived prosocial taxation positively predicted (1) favorable views of taxation, (2) satisfaction paying taxes, and (3) willingness to pay taxes. Moreover, we examined whether these relationships remained while controlling for prosocial tendencies in general and various socio-demographic variables. See S1 Tables for full correlation and regression information.

First, using a series of bivariate Pearson’s correlations, we investigated the relationship between perceived prosocial taxation and our key outcome measures. Analyses supported our predictions and revealed that perceived prosocial taxation was positively correlated with how positively participants viewed their taxes, *r*(499) = .62, *p* < 0.001, how satisfied participants felt paying their taxes, *r*(499) = .60, *p* < 0.001, and how willing participants were to pay their taxes, *r*(499) = .55, *p* < 0.001.

Importantly, a series of multiple linear regressions revealed that these findings remained when controlling for general prosocial tendencies, household income, political attitudes, age, and gender. Specifically, the first linear regression demonstrated that perceived prosocial taxation positively predicted how participants viewed their taxes (β = .62, *t* = 16.20, *p <* 0.001), while prosocial orientation (β = .003, *t* = 0.09, *p =* 0.93), gender (β = -.03, *t* = -0.73, *p =* 0.46), and political attitudes (β = -.01, *t* = -0.16, *p =* 0.87) did not. Meanwhile, older (β = -.09, *t* = -2.42, *p =* 0.02) and wealthier individuals (β = -.09, *t* = -2.50, *p =* 0.01) reported more negative perceptions of taxation.

A second linear regression showed that perceived prosocial taxation predicted satisfaction paying taxes (β = .59, *t* = 15.30, *p <* 0.001), but prosocial orientation (β = .02, *t* = 0.45, *p =* 0.65), age (β = -.04, *t* = -1.09, *p =* 0.28), gender (β = -.07, *t* = -1.87, *p =* 0.06), political attitudes (β = -.01, *t* = -0.36, *p =* 0.72), and income (β = -.02, *t* = -0.41, *p =* 0.69) did not.

Finally, a third linear regression revealed that perceived prosocial taxation (β = .53, *t* = 13.13, *p <* 0.001) predicted willingness to pay taxes but prosocial orientation (β = .05, *t* = 1.14, *p =* 0.25), gender (β = -.03, *t* = -0.80, *p =* 0.42), age (β = -.02, *t* = -0.56, *p =* 0.58), political attitudes (β = -.03, *t* = -0.85, *p =* 0.39), and income (β = .02, *t* = 0.60, *p =* 0.55) did not. Thus, people who recognize the positive impact that taxation can have on society are more supportive of taxation, above and beyond their general prosocial tendencies and various socio-demographic characteristics.

While Study 1 provides a first look at this question in a large and nationally representative sample of Americans, we were not able to rule out the possibility that perceived prosocial taxation predicts support for taxation because it implies that tax dollars are being used properly and not wasted. Indeed, it is possible that simply seeing tax dollars being put to use predicts greater support, even if tax dollars are not being used to help other citizens (e.g., [17]). To address this alternative explanation, participants in Studies 2 and 3 reported their perceptions of tax utility, allowing us to examine whether perceived prosocial taxation predicts support for taxes above and beyond perceptions that tax dollars are being well used as well as other important controls.

## Study 2

### Material and methods

#### Participants

One-hundred and thirteen psychology undergraduate students from Simon Fraser University (*M*_*age*_ = 20.19, *SD =* 3.69, 23% male, 75.2% female, 1.8% selected “other” or did not respond) were recruited to participate in a survey in exchange for course credit between September 2015 and April 2016.

#### Procedures

Participants signed up for a study on problem solving and, upon arrival in the lab, were asked to complete an easy puzzle task. The task was designed to be simple so that all participants would earn the full payment ($5 CDN). Participants signed a receipt acknowledging they received $5 and were told that they would be taxed 20% ($1 CDN) as part of a new lab initiative to support the local student government (“Simon Fraser University Student Society” or “SFSS”; see [14] for similar methods).

After completing multiple measures not pertinent to the present research, participants responded to several questions measuring support for the SFSS tax. Specifically, participants responded to the questions: “How much did you enjoy paying the SFSS tax?”, “To what extent do you think that the SFSS tax is worthwhile?”, and finally, “To what extent do you think that we should continue collecting the SFSS tax in our experiments?” using Likert scales from 1 (Not at all) to 11 (Extremely). Participants also reported their feelings of perceived prosocial taxation by answering, “To what extent do you think that your SFSS tax will have a positive impact on others (e.g., SFU students)?” and their perceptions of tax utility in general by rating “To what extent do you think that your SFSS tax will be put to good use?” Following this, participants reported their demographic information, including their age and gender (1 = Male, 2 = Female, 3 = Other). Note that two participants (one who identified their gender as “other” and another who did not identify their gender) were excluded from regressions controlling for gender due to the lack of sufficient observations for these gender categories.

### Results

We examined whether perceived prosocial taxation was positively associated with (1) enjoyment paying the tax, (2) perception of the tax as worthwhile, and (3) views that the tax should continue. We also examined whether these associations remained while controlling for perceptions of tax utility. See S2 Tables for full correlation and regression tables.

First, using a series of bivariate Pearson’s correlations, we investigated the relationship between perceived prosocial taxation and our key outcome measures. Supporting our predictions, analyses revealed that perceived prosocial taxation was positively associated with enjoyment of the tax, *r*(111) = .54, *p <* 0.001, perception of the tax as being worthwhile *r*(111) = .70, *p <* 0.001, and views that the lab tax should continue, *r*(111) = .56, *p <* 0.001.

A series of multiple regressions showed that these findings remained while controlling for perceived tax utility, age, and gender. Specifically, when perceived prosocial taxation, perceptions of tax utility, and demographic controls were used to predict people’s enjoyment of the tax, perceived prosocial taxation was the only significant predictor (β = .40, *t* = 3.38, *p =* 0.001). General perceptions of tax utility (β = .22, *t* = 1.87, *p =* 0.06), age (β = .06, *t* = 0.71, *p =* 0.48), and gender (β = -.07, *t* = -0.85, *p =* 0.40) did not predict enjoyment paying the tax.

A second multiple regression model revealed that perception of the tax as being worthwhile was predicted by both perceived prosocial taxation (β = .35, *t* = 3.94, *p <* 0.001) and tax utility (β = .49, *t* = 5.45, *p <* 0.001) while controlling for age (β = -.02, *t* = -0.26, *p =* 0.80) and gender (β = -.05, *t* = -0.80, *p =* 0.43). Finally, a third multiple regression model indicated that support for tax continuation was predicted by both perceived prosocial taxation (β = .24, *t* = 2.24, *p =* 0.03) and tax utility (β = .46, *t* = 4.19, *p <* 0.001) when controlling for age (β = .00, *t* = -0.001, *p =* 0.99) and gender (β = -.14, *t* = -1.85, *p =* 0.07). Thus, perceived prosocial taxation predicted support for taxation above and beyond general perceptions of tax utility and several basic demographic variables.

Study 2 offered additional support for the hypothesis that perceived prosocial taxation is associated with greater support for taxation, even when controlling for general perceptions that tax dollars are being put to good use. While promising, our investigation thus far has focused on self-reported behavior of taxation attitudes. Therefore, in Study 3, we attempted to replicate the results of Studies 1–2 and examine whether recognizing the prosocial benefits of taxation predicts larger financial contributions in a real tax-like transfer.

## Study 3

### Material and methods

#### Participants

Two-hundred and ninety-two psychology undergraduate students from Simon Fraser University (*M*_*age*_ = 19.27, *SD =* 3.12, 26% male, 74% female) were recruited in exchange for course credit between January and April 2016.

#### Procedures

Participants came into the lab individually and completed a puzzle task. Each question answered correctly allowed the participant to earn $.50 CDN. With 10 questions, participants could earn up to $5 CDN. However, instead of enforcing a 20% tax to the Simon Fraser Student Society (SFSS) as we did in Study 2, participants were reminded of the services that SFSS provides (e.g., student newspaper, student radio station) and allowed to choose how much, if any, of their earnings they wanted to contribute as a tax to SFSS. The amount participants chose to donate was divided by their earnings in the puzzle task and multiplied by 100 to create a “percentage of earnings donated” variable. Participants also completed the same support for taxation measures used in Study 2, where they indicated their enjoyment paying the tax, perceptions of whether the tax was worthwhile, and whether the tax should continue. Finally, participants reported their demographic information (age and gender).

### Results

Similar to Study 2, we hypothesized that greater perceived prosocial taxation would be positively associated with (1) enjoyment paying the tax (2) perception of the tax as worthwhile, (3) support for the tax to continue, and (4) larger financial contributions to SFSS. We also investigated whether these findings remained while controlling for tax utility and several demographics. See S3 Tables for full correlation and regression tables.

Using a series of bivariate Pearson’s correlations, we investigated the relationship between perceived prosocial taxation, participants’ feelings about the tax, and amount contributed to SFSS. Consistent with our predictions and previous studies, perceived prosocial taxation was positively associated with enjoyment of the tax, *r*(290) = .25, *p <* 0.001, perception of the tax as being worthwhile *r*(290) = .34, *p <* 0.001, support for the tax to continue, *r*(290) = .23, *p <* 0.001, and larger financial contributions to SFSS, *r*(290) = .19, *p =* 0.001.

As in Study 2, we used multiple regressions to examine whether the findings reported above remained while controlling for perceived tax utility, gender, and age. Analyses revealed that both perceived prosocial taxation (β = .20, *t* = 3.59, *p =* 0.001) and tax utility (β = .23, *t* = 4.10, *p <* 0.001) predicted enjoyment of taxation, but age (β = -.01, *t* = -0.12, *p =* 0.90) and gender did not (β = .09, *t* = 1.64, *p =* 0.10).

Similarly, perception of the tax as worthwhile was predicted by both perceived prosocial taxation (β = .30, *t* = 5.47, *p <* 0.001) and tax utility (β = .25, *t* = 4.60, *p <* 0.001), but not by age (β = -.01, *t* = -0.18, *p =* 0.86) or gender (β = .07, *t* = 1.28, *p =* 0.20).

Agreement that the tax should continue was predicted by perceived prosocial taxation (β = .22, *t* = 3.71, *p <* 0.001) but not by tax utility (β = .11, *t* = 1.91, *p =* 0.06), age (β = .04, *t* = 0.61, *p =* 0.55), or gender (β = -.03, *t* = -0.47, *p =* 0.64).

Finally, the percentage of one’s study earnings given to SFSS was predicted by perceived prosocial taxation (β = .18, *t* = 2.99, *p <* 0.01) and by tax utility (β = .12, *t* = 2.04, *p =* 0.04), but not by age (β = .03, *t* = 0.49, *p =* 0.63) or gender (β = -.003, *t* = -0.05, *p =* 0.96). Thus, perceived prosocial taxation predicted support for taxation above and beyond perceptions of the tax as useful and two basic demographic variables.

Among a larger student sample, Study 3 replicates the link between perceived prosocial taxation and support for taxation, even while controlling for perceptions that tax dollars are being put to good use and important demographic controls. Moreover, Study 3 reveals that perceived prosocial taxation is associated with larger tax-like contributions to SFSS.

Studies 1–3 probe the relationship between perceived prosocial taxation and support for taxes in a nationally representative sample of Americans and two samples of Canadian students. Readers may wonder whether the association between perceived prosocial taxation and support for taxes extends to other cultures and contexts outside North America. To explore the generality of this relationship, we analyzed data from several waves of the World Values Survey (WVS), a large questionnaire administered to nearly half a million respondents in more than 100 countries. While the WVS does not include direct measures of perceived prosocial taxation or support for taxes, it does tap into relevant constructs, providing a first look at this association in wide-ranging samples drawn from many countries.

## Study 4

### Material and methods

#### Participants

The data come from six waves of the World Values Survey (WVS), spanning the first wave in 1981–1984 to the sixth wave in 2010–2014 [18]. The sample consists of 474,000 respondents in 107 countries who provided responses to our key measures.

#### Measures

The WVS asks respondents whether it is ever justifiable to cheat on taxes (question f116). We used this question as our measure of support for taxation. Respondents who answer that it is never justifiable to cheat on taxes are considered to be tax compliers.

Because the WVS does not directly probe respondents’ levels of perceived prosocial taxation, we triangulate on this construct by using two measures of taxpayer beliefs that their taxes would be used in prosocial ways. The WVS asks respondents separately about the confidence they have in the government (e069_11) and in the civil service (e069_08). Those who answer ‘a great deal’ (i.e., provide the highest rating among the four response options) to each question are considered to have a basis for optimism that taxes collected would be spent in prosocial ways. About 40,000 respondents had a great deal of confidence in government and 39,000 a great deal of confidence in the civil services. Only 16,000 had a great deal of confidence in both. Consistent with our earlier results, we would expect that respondents would be more likely to be tax compliant if they had more confidence in government and the civil services. These effects were estimated with and without controlling for a number of other variables that might be expected to influence tax compliance, including age, income, marital status, gender, and employment status.

#### Procedure

The tax compliance effects of confidence in government and the civil services are estimated by probit regressions showing the probability that an individual will be tax compliant. In all cases we include country fixed effects to explain differences in tax compliance within the taxpayer’s society, which is consistent with our approach in Studies 1–3. Errors are assumed to be clustered at the country level. We assess the extent to which these effects differ by country grouping, and estimate them with and without controlling for other individual-level variables that might be expected to also influence tax compliance. For WVS data, see http://www.worldvaluessurvey.org/WVSDocumentationWVL.jsp.

### Results

Before testing our main hypothesis, we examined if and how tax compliance varied across countries. The WVS data suggest that the norm of tax compliance is fairly universal. Those who think that it is never justifiable to cheat on taxes are in a majority in 85 of the 107 countries, over 40% in all but seven countries, and over 30% in all countries. Tax compliers comprise 59% of the 216,000 respondents in the 34 industrial countries of the Organization for Economic Cooperation and Development (OECD) and 61% of the 258,000 respondents from 73 other countries. See Table I in S4 Tables for national tax compliance differences and Tables A-H in S4 Tables for the regression tables.

To test our central hypothesis, we examined whether respondents reporting a great deal of support in their government and civil service were more likely to say that it is never acceptable to cheat on taxes. Supporting this possibility, we find that those with a great deal of confidence in their governments and civil services are more likely to be tax compliant. The probability of being tax compliant is estimated to be 14.2% higher (β = .14, *t* = 5.96, *p* < .001) for those with a great deal of confidence in government, and 15.6% higher (β = .16, *t* = 6.84, *p* < .001) for those with a great deal of confidence in the civil services. Since these results come from the same estimation, it can be seen that tax compliance is therefore almost 30% higher (sum of β = .298) for those with a great deal of confidence in both government and the civil services. Although these estimates are material in size and highly significant in statistical terms, aided by the very large samples, the overall probit regression only explains about 5% of the range of answers across individuals, and most of this is provided by the country fixed effects.

To examine the global robustness for these estimates, we repeated the analysis separately for the OECD and non-OECD countries separately. Interestingly, the coefficient sizes are significantly larger for the industrial countries, but respondents in those countries are also significantly less likely to have a great deal of confidence in either their governments or the civil services. When the results are calculated as standardized betas, these two effects are naturally offsetting, to give effects of .02 (*t* = 9.96) and .04 (*t* = 19.79) for the OECD countries, and .03 (*t* = 14.51) and .02 (*t* = 8.37) for the non-OECD countries (*p* < 0.001 for all). When we look specifically at the United States and Canada, the countries providing the data for our earlier studies, we see that they are more tax compliant than most other countries (68% and 66% for the United States and Canada, respectively). In both cases, those who have great deal of confidence in government are few in number, about 3% in both cases, and have weaker links to tax compliance. Those with a great deal of confidence in the civil services are two or three times more numerous, and are significantly more likely to be tax compliant. The standardized betas for civil service confidence are .04 (*t* = 4.07, *p* < 0.001) for the United States, and .06 (*t* = 4.87, *p* < 0.001) for Canada. These are similar to the results for the other OECD countries.

Taken together, the WVS results suggest that taxpayer compliance is relatively high all over the world, and associated with measures indicating taxpayer belief that their taxes are being effectively spent for public purposes. The effect sizes, as measured by standardized betas, are of the same order of magnitude all over the world. To the extent that there is a difference between the OECD and other countries, it is that in the OECD countries high confidence in government is more prevalent than is trust in the civil services, and is a more powerful predictor of taxpayer compliance. Confidence in the civil services is higher than in government in the OECD countries (although still only half as great as in the non-OECD countries), but has a more robust link to taxpayer compliance, as shown by the standardized betas.

Adding a range of control variables shows that females are more likely than males to be tax compliant (+.12, *t =* 9.68), as are those who are older (.01 per year of age, *t =* 16.99), married (+.06, *t* = 5.55), or with college or higher education (+.05, *t =* 2.54) while those with higher incomes (-.02 per income decile, *t =* 5.86) and the unemployed (-.04, *t* = -1.91) are less tax compliant (all effects are highly significant, *p* < 0.01, except for unemployment, with *p* = 0.06). In these controlled models, the effects of confidence are only slightly lower (0.11 and 0.14 for government and the civil services, respectively) and remain highly significant (*t* = 4.34 and *t* = 6.33 respectively).

## General discussion

Four correlational studies suggest that taxation is seen in a more positive light when taxpayers believe that their contributions are effectively helping others. Importantly, this finding remains when controlling for respondents’ overall prosocial orientation, general perceptions of tax utility, and various socio-economic demographics. Specifically, in Study 1, we found that individuals who thought their taxes assisted others reported more positive perceptions of taxation and a greater willingness to pay their taxes. Study 2 replicated and extended these findings by demonstrating that the more that students thought their tax dollars would be beneficial for others, the more they enjoyed paying a lab tax, considered the tax worthwhile, and expressed support for the lab tax to continue. Study 3 revealed that the more that participants thought their tax dollars would help others, the more positively they viewed the tax, supported its continuation, and gave a larger percentage of their actual earnings to the student tax. Finally, data from over 100 countries in the World Values Survey show that people expressing a high level of confidence in their government and civil service–thereby indicating some optimism that their taxes will be used in prosocial ways–are more likely to state that it is never justifiable to cheat on taxes. These findings offer a new and optimistic perspective on taxation. Rather than seeing taxes as an unwelcome payment, people may hold more positive views and be more willing to contribute if they believe their contribution benefits other citizens in society.

### Theoretical applications

Our results are consistent with past findings demonstrating that prosocial inclinations are associated with increased support for taxation [12–14]. However, while past work has observed a link between generalized concern for others and tax compliance, the present research investigates whether people’s perception of the tax itself impacts their support for taxation, regardless of their personal prosocial inclinations. As such, this work adds to the growing body of research examining whether and how social norms and interactions influence tax evasion and compliance [19–22]. Moreover, this is the first research that we are aware of to find that people who recognize the benefits of taxation for others actually contribute more money in a tax-like situation.

### Practical applications

Our findings suggest that people who recognize the prosocial benefits of taxation may be more supportive of taxation, and be more tax compliant. For this reason, governments should consider making information regarding the prosocial impact of taxation more salient. For instance, governments could advertise that tax-funded highway construction improves the commute of thousands of citizens daily, or that tax dollars are used to provide warm breakfast meals for thousands of kids across the country that help them focus at school. These examples highlight ways in which tax dollars are used to help others and may make tax support more likely. Indeed, if future research using an experimental paradigm shows that reminding people of the prosocial benefits of taxation can cause increased tax compliance, giving taxpayers information highlighting the benefits of taxation for others could encourage tax compliance on a larger scale, potentially reducing the extent and costs of tax aversion and evasion.

### Limitations and future directions

While this work offers new insight into what factors are associated with greater support for taxation, it is important to note that the present studies used correlational designs and therefore cannot address questions of causality. As such, future research should explore whether and how perceptions of prosocial taxation can be bolstered and whether this leads to increased support for taxation. This may be a more powerful and robust way to test whether asking people to consider the prosocial benefits of taxation increases support for taxation.

In addition, two of the present studies surveyed convenience samples of psychology undergraduate students, raising questions about whether results apply to the broader population. However, we observed a consistent pattern of results in a national panel of over 500 Americans and converging results across 107 nations worldwide, supporting the generality of these findings. Future research could investigate in more detail if and how the relationship varies across different samples and societies.

To this end, results like these are surely related to the social norms of the countries where the studies are conducted. Study 1 involves a survey population of residents of the United States, while Studies 2 and 3 involve undergraduates at a Canadian university, most of whom are resident Canadians. The broad transferability of our results to other countries, where public expenditures and tax systems have differing degrees of effectiveness, integrity, and acceptability is suggested by Study 4, based on data from 107 countries. For other international experiments in this direction, see [21–24].

It is worth noting that the correlations observed between perceived prosocial taxation and support for taxation in Study 2 were much larger than those in Study 3, despite the near identical design. We are not certain about the reason for this difference, but we have considered two, non-conflicting possibilities. The first is an important procedural difference between the two studies. Specifically, participants in Study 3 chose how much of their earnings they would like to contribute to the lab tax, while participants in Study 2 were required to pay 20%. Thus, participants in Study 3 gave approximately three times more (i.e. an average of 60%) to the student union society than participants in Study 2. A second explanation may be the difference in sample size, with Study 3 (*N* = 292) being over twice that of Study 2 (*N* = 113). Although findings in both studies aligned with predictions, the smaller association detected in Study 3 may be a more robust estimate of the true effect size because larger sample sizes often produce more reliable approximations [25]. Ideally future research will provide further insight into the size of associations between perceived prosocial taxation and support for taxes.

Finally, while we found that perceived prosocial impact was associated with positive perceptions of taxation, even when controlling for perceived tax utility and general prosocial orientation, we used single item measures of these constructs. Because these may not be the most reliable measures, future research should re-examine the relationship between prosocial orientation, prosocial impact, and perceived tax utility using more robust multi-item measures.

## Conclusions

People who recognize the benefits of taxation for others also report greater support for taxation, showed greater willingness to pay taxes, expressed greater support for the tax to continue, contributed more money in a tax-like donation, and expressed a lower likelihood of cheating on their taxes. Results remain while controlling for participants’ general prosocial inclinations, perceived tax utility, as well as important socio-demographic variables. These findings offer an optimistic perspective on taxation, suggesting that people may be more willing to pay their taxes if they believe their contribution benefits others.

## Supporting information

S1 Tables Supporting information 1 for “Prosocial perceptions of taxation predict support for taxes”. (DOCX)

S2 Tables Supporting information 2 for “Prosocial perceptions of taxation predict support for taxes”. (DOCX)

S3 Tables Supporting information 3 for “Prosocial perceptions of taxation predict support for taxes”. (DOCX)

S4 Tables Supporting information 4 for “Prosocial perceptions of taxation predict support for taxes”. (DOCX)

## References

[1] CNN. Poll: Most Americans say tax dollars are wasted. CNN. 15 Apr 2010. http://www.cnn.com/2010/POLITICS/04/15/poll.wasted.taxes/index.html

[2] Sussman AB, Olivola CY. Axe the tax: Taxes are disliked more than equivalent costs. JMR. 2011; 48, S91–S101.

[3] OECD. Revenue statistics: Comparative tables; 2019. Database: OECD Tax Statistics. 10.1787/data-00262-en

[4] Mettler S. The submerged state: How invisible government policies undermine American democracy. Chicago, IL: University of Chicago Press; 2019.

[5] Alm J. Measuring, explaining, and controlling tax evasion: Lessons from theory, experiments, and field studies. Int Tax and Public Finance. 2012; 19, 54–77.

[6] Cobham A, Janský P. Measurement of illicit financial flows. UNODC-UNCTAD expert consultation on the SDG Indicator on illicit financial flows: Background paper prepared for UNCTAD: Benefits and Costs of the IFF Targets. 2017; 187–238.

[7] Penner LA, Dovidio JF, Piliavin JA, Schroeder DA. Prosocial behavior: Multilevel perspectives. Annu Rev Psychol. 2005; 56, 365–392. 10.1146/annurev.psych.56.091103.070141 15709940

[8] Klein N, Grossman I, Uskul AK, Kraus A, Epley N. It pays to be nice, not really nice: Asymmetric evaluations of prosociality across seven cultures. Judgm Decis Mak. 2015; 10, 355–364.

[9] Henrich J, Henrich N. Culture, evolution and the puzzle of human cooperation. Cogn Syst Res. 2006; 7, 220–245.

[10] Aknin LB, Barrington-Leigh CP, Dunn EW, Helliwell JF, Burns J, Biswas-Diener R, et al Prosocial spending and well-being: Cross-cultural evidence for a psychological universal. J Pers Soc Psychol. 2013; 104, 635–652. 10.1037/a0031578 23421360

[11] Aknin L. Whillans AV, Norton MI, Dunn EW. Happiness and prosocial behaviour: An evaluation of the evidence. In: Helliwell J, Layard R, Sachs K, editors. World Happiness Report 2019, New York: Sustainable Development Solutions Network; 2019.

[12] Brizi A, Giacomantonio M, Schumpe BM, Mannetti L. Intention to pay taxes or to avoid them: The impact of social value orientation. J Econ Psychol. 2015; 50, 22–31.

[13] Andriani L. Tax morale and prosocial behaviour: Evidence from a Palestinian survey. Cambridge J Econ. 2015; 40, 821–841.

[14] Whillans AV, Wispinski NJ, Dunn EW. Seeing wealth as a responsibility improves attitudes towards taxation. J Econ Behav Organ. 2016; 127, 146–154.

[15] Grant AM, Campbell EM, Chen G, Cottone K, Lapedis D, Lee K. Impact and the art of motivation maintenance: The effects of contact with beneficiaries on persistence behavior. Organ Behav Hum Decis Process. 2007; 103, 53–67.

[16] Small DA, Loewenstein G. Helping a victim or helping the victim: Altruism and identifiability. J Risk Uncertain. 2003; 26, 5–16.

[17] Barone G, Mocetti S. Tax morale and public spending inefficiency. Int Tax and Public Finance. 2011; 18, 724–749.

[18] World Values Survey. (2019). On-line data analysis. Retrieved in October 2019, from www.worldvaluessurvey.com

[19] Andreoni J, Erard B, Feinstein J. Tax compliance. J Econ Lit. 1998; 36(2), 818–860.

[20] Fortin B, Lacroix G, Villeval MC. Tax evasion and social interactions. J Public Econ. 2007; 91, 2089–2112.

[21] Lefebvre M, Pestieau P, Riedl A, Villeval MC. Tax evasion and social information: An experiment in Belgium, France, and the Netherlands. Int Tax and Public Finance. 2015; 22, 401–425.

[22] Bobek DD, Roberts RW, Sweeney JT. The social norms of tax compliance: Evidence from Australia, Singapore, and the United States. J Bus Ethics. 2007; 74, 49–64.

[23] Ferrer-i-Carbonell A, Gërxhani K. Tax evasion and well-being: A study of the social and institutional context in Central and Eastern Europe. Eur J Polit Econ. 2016; 45, 149–159.

[24] Kettle S, Hernandez M, Ruda, S, Sanders M. Behavioral interventions in tax compliance: Evidence from Guatemala. In Policy Research Working Paper 7690. Washington, DC: World Bank; 2016.

[25] Fraley RC, Vazire S. The N-pact factor: Evaluating the quality of empirical journals with respect to sample size and statistical power. PloS one. 2014, 10, e109019. 10.1371/journal.pone.0109019 PMC4189949 25296159

