# Supplementary material for: Prosocial perceptions of taxation predict support for taxes
Source: PLoS One. 2019 Nov 26;14(11):e0225730. doi: 10.1371/journal.pone.0225730 (PMC6879120; doi:10.1371/journal.pone.0225730)
Supplement: S1 Tables — (DOCX) [file pone.0225730.s001.docx]

Supporting Information 1 for “*Prosocial perceptions of taxation predict support for taxes*”

by Thornton, Aknin, Branscombe, and Helliwell

**Table A.** Correlations between perceived prosocial taxation and perceptions of taxation in Study 1 (*N* = 501).

|  | Perceived Prosocial Taxation (1-7) | Feel positively about tax  (1-100) | Satisfied with tax  (1-100) | Willing to pay taxes  (1-100) | Mean (SD) |
| --- | --- | --- | --- | --- | --- |
| Perceived Prosocial Taxation | 1 |  |  |  | 4.91 (1.46) |
| Feel positively about tax | .62* | 1 |  |  | 54.71 (27.91) |
| Satisfied with tax | .60* | .68* | 1 |  | 56.67 (31.21) |
| Willing to pay taxes | .55* | .46* | .66* | 1 | 71.72 (28.98) |

*Note*: * indicates *p* < 0.05

|  | Std. β | β | *SE* | *t* | *p* |
| --- | --- | --- | --- | --- | --- |
| Perceived Prosocial Taxation | .62 | 11.89 | 0.73 | 16.20 | < 0.001 |
| General Prosocial Inclination | .003 | 0.09 | 0.91 | 0.09 | 0.93 |
| Gender | -.03 | -1.48 | 2.02 | -0.73 | 0.46 |
| Politics | -.01 | -0.09 | 0.55 | -0.16 | 0.87 |
| Age | -.09 | -0.15 | 0.06 | -2.42 | 0.02 |
| Income | -.09 | -1.24 | 0.49 | -2.50 | 0.01 |

**Table B.** Perceived prosocial taxation predicts more positive feelings towards taxes while controlling for general prosocial inclination, age, gender, political orientation, and income in Study 1 (*N* = 501)

Adjusted *R^2^* = .38

**Table C.** Perceived prosocial taxation predicts higher satisfaction with paying taxes while controlling for general prosocial inclination, age, gender, political orientation, and income in Study 1 (*N* = 501).

|  | Std. β | β | *SE* | *t* | *p* |
| --- | --- | --- | --- | --- | --- |
| Perceived Prosocial Taxation | .59 | 12.76 | 0.83 | 15.30 | < 0.001 |
| General Prosocial Inclination | .02 | 0.47 | 1.04 | 0.45 | 0.65 |
| Age | -.04 | -0.08 | 0.07 | -1.09 | 0.28 |
| Gender | -.07 | -4.29 | 2.29 | -1.87 | 0.06 |
| Politics | -.01 | -0.22 | 0.62 | -0.36 | 0.72 |
| Income | -.02 | -0.23 | 0.56 | -0.41 | 0.69 |

Adjusted *R^2^* = .36

|  | Std. β | β | *SE* | *t* | *p* |
| --- | --- | --- | --- | --- | --- |
| Perceived Prosocial Taxation | .53 | 10.66 | 0.81 | 13.13 | < 0.001 |
| General Prosocial Inclination | .05 | 1.15 | 1.01 | 1.14 | 0.25 |
| Gender | -.03 | -1.79 | 2.23 | -0.80 | 0.42 |
| Age | -.02 | -0.04 | 0.07 | -0.56 | 0.58 |
| Politics | -.03 | -0.51 | 0.60 | -0.85 | 0.39 |
| Income | .02 | 0.33 | 0.55 | 0.60 | 0.55 |

**Table D.** Perceived prosocial taxation predicts greater willingness to pay taxes while controlling for general prosocial inclination, age, gender, political orientation, and income in Study 1 (*N* = 501).

Adjusted *R^2^* = .30
