# Supplementary material for: Prosocial perceptions of taxation predict support for taxes
Source: PLoS One. 2019 Nov 26;14(11):e0225730. doi: 10.1371/journal.pone.0225730 (PMC6879120; doi:10.1371/journal.pone.0225730)
Supplement: S2 Tables — (DOCX) [file pone.0225730.s002.docx]

Supporting Information 2 for “*Prosocial perceptions of taxation predict support for taxes*”

by Thornton, Aknin, Branscombe, and Helliwell

**Table A.** Correlations between perceived prosocial taxation and perceptions of taxation in Study 2 (*N* = 113).

|  | Perceived Prosocial Taxation  (1-11) | Enjoyment of Tax  (1-11) | Tax as worthwhile  (1-11) | Tax should continue  (1-11) | Mean (SD) |
| --- | --- | --- | --- | --- | --- |
| Perceived Prosocial Taxation | 1 |  |  |  | 7.42 (2.36) |
| Enjoyment of Tax | .54* | 1 |  |  | 6.84 (2.12) |
| Tax as worthwhile | .70* | .66* | 1 |  | 7.80 (2.11) |
| Tax should continue | .56* | .65* | .73* | 1 | 8.01 (2.43) |

*Note*: * indicates *p* < 0.05

|  | Std. β | β | *SE* | *t* | *p* |
| --- | --- | --- | --- | --- | --- |
| Perceived Prosocial Taxation | .40 | 0.36 | 0.11 | 3.38 | 0.001 |
| Perceived Tax Utility | .22 | 0.23 | 0.12 | 1.87 | 0.06 |
| Age | .06 | 0.03 | 0.05 | 0.71 | 0.48 |
| Gender | -.07 | -0.35 | 0.41 | -0.85 | 0.40 |

**Table B**. Perceived prosocial taxation predicts greater enjoyment paying the SFSS tax while controlling for perceived tax utility, age, and gender in Study 2 (*N* = 113).

Adjusted *R*^2^ = .30

**Table C.** Perceived prosocial taxation predicts views that the SFSS tax is worthwhile while controlling for perceived tax utility, age, and gender in Study 2 (*N* = 113).

|  | Std. β | β | *SE* | *t* | *p* |
| --- | --- | --- | --- | --- | --- |
| Perceived Prosocial Taxation | .35 | 0.31 | 0.08 | 3.94 | < 0.001 |
| Perceived Tax Utility | .49 | 0.50 | 0.09 | 5.45 | < 0.001 |
| Age | -.02 | -0.01 | 0.04 | -0.26 | 0.80 |
| Gender | -.05 | -0.25 | 0.31 | -0.80 | 0.43 |

Adjusted *R^2^* = .59

**Table D.** Perceived prosocial taxation predicts greater willingness to continue paying the SFSS tax while controlling for perceived tax utility, age, and gender in Study 2 (*N* = 113).

|  | Std. β | β | *SE* | *t* | *p* |
| --- | --- | --- | --- | --- | --- |
| Perceived Prosocial Taxation | .24 | 0.25 | 0.11 | 2.24 | 0.03 |
| Perceived Tax Utility | .46 | 0.54 | 0.13 | 4.19 | < 0.001 |
| Age | .00 | < -0.001 | 0.05 | -0.001 | 0.99 |
| Gender | -.14 | -0.80 | 0.44 | -1.85 | 0.07 |

Adjusted *R*^2^ = .40
