# Supplementary material for: Prosocial perceptions of taxation predict support for taxes
Source: PLoS One. 2019 Nov 26;14(11):e0225730. doi: 10.1371/journal.pone.0225730 (PMC6879120; doi:10.1371/journal.pone.0225730)
Supplement: S3 Tables — (DOCX) [file pone.0225730.s003.docx]

Supporting Information 3 for “*Prosocial perceptions of taxation predict support for taxes*”

by Thornton, Aknin, Branscombe, and Helliwell

|  | Perceived Prosocial Taxation  (1-11) | Enjoyment of Tax  (1-11) | Tax as worthwhile  (1-11) | Tax should continue  (1-11) | % Contributed to SFSS | Mean (SD) |
| --- | --- | --- | --- | --- | --- | --- |
| Perceived Prosocial Taxation | 1 |  |  |  |  | 6.71 (5.35) |
| Enjoyment of Tax | .25* | 1 |  |  |  | 5.96 (2.38) |
| Tax as worthwhile | .34* | .63* | 1 |  |  | 6.52 (2.33) |
| Tax should continue | .23* | .58* | .67* | 1 |  | 6.78 (2.47) |
| % Contributed to SFSS | .19* | .47* | .39* | .37* | 1 | .60 (0.42) |

**Table A**. Correlations between perceived prosocial taxation and perceptions of taxation in Study 3 (*N* = 291).

*Note*: * indicates *p* < 0.05

.

**Table B.** Perceived prosocial taxation predicts greater enjoyment paying the SFSS tax while controlling for perceived tax utility, age, and gender in Study 3 (*N* = 291).

|  | Std. β | β | *SE* | *t* | *p* |
| --- | --- | --- | --- | --- | --- |
| Perceived Prosocial Taxation | .20 | 0.09 | 0.03 | 3.59 | < 0.001 |
| Perceived Tax Utility | .23 | 0.07 | 0.02 | 4.10 | < 0.001 |
| Age | -.01 | -0.01 | 0.05 | -0.12 | 0.90 |
| Gender | .09 | 0.46 | 0.28 | 1.64 | 0.10 |

Adjusted *R*^2^ = .11

|  | Std. β | β | *SE* | *t* | *p* |
| --- | --- | --- | --- | --- | --- |
| Perceived Prosocial Taxation | .30 | 0.13 | 0.02 | 5.47 | < 0.001 |
| Perceived Tax Utility | .25 | 0.07 | 0.02 | 4.60 | < 0.001 |
| Age | -.01 | -0.01 | 0.05 | -0.18 | 0.86 |
| Gender | .07 | 0.34 | 0.27 | 1.28 | 0.20 |

**Table C.** Perceived prosocial taxation predicts higher perceptions of the SFSS tax as worthwhile while controlling for perceived tax utility, age, and gender in Study 3 (*N* = 292).

Adjusted *R*^2^ = .17

**Table D.** Perceived prosocial taxation predicts greater willingness to continue paying the SFSS tax while controlling for perceived tax utility, age, and gender in Study 3 (*N* = 292).

|  | Std. β | β | *SE* | *t* | *p* |
| --- | --- | --- | --- | --- | --- |
| Perceived Prosocial Taxation | .22 | 0.10 | 0.03 | 3.71 | < 0.001 |
| Perceived Tax Utility | .11 | 0.03 | 0.02 | 1.91 | 0.06 |
| Age | .04 | 0.03 | 0.05 | 0.61 | 0.55 |
| Gender | -.03 | -0.14 | 0.30 | -0.47 | 0.64 |

Adjusted *R^2^* = .05

**Table E.** Perceived prosocial taxation predicts amount of money contributed to the SFSS tax while controlling for perceived tax utility, age, and gender in Study 3 (*N* = 292).

|  | Std. β | β | *SE* | *t* | *p* |
| --- | --- | --- | --- | --- | --- |
| Perceived Prosocial Taxation | .18 | 0.01 | 0.01 | 2.99 | < 0.01 |
| Perceived Tax Utility | .12 | 0.01 | 0.003 | 2.04 | 0.04 |
| Age | .03 | 0.004 | 0.01 | 0.49 | 0.63 |
| Gender | -.003 | -0.003 | 0.05 | -0.05 | 0.96 |

Adjusted *R*^2^ = .04
