# Supplementary material for: Prosocial perceptions of taxation predict support for taxes
Source: PLoS One. 2019 Nov 26;14(11):e0225730. doi: 10.1371/journal.pone.0225730 (PMC6879120; doi:10.1371/journal.pone.0225730)
Supplement: S4 Tables — (DOCX) [file pone.0225730.s004.docx]

Supporting Information 4 for “*Prosocial perceptions of taxation predict support for taxes*”

by Thornton, Aknin, Branscombe, and Helliwell

|  | β | *SE* | *t* | *p* |
| --- | --- | --- | --- | --- |
| Confidence in Government | 0.14 | 0.02 | 5.96 | < 0.001 |
| Confidence in Civil Services | 0.16 | 0.02 | 6.84 | < 0.001 |

**Table A.** Probit regression using confidence in government and in civil services to predict tax compliance without controls (*N* = 420,346).

*Note. R^2^ = 0.05. Country fixed effect coefficients are estimated but not reported.*

**Table B.** Probit regression using confidence in government and in civil services to predict tax compliance with controls (*N* = 301,525).

|  | β | *SE* | *t* | *p* |
| --- | --- | --- | --- | --- |
| Confidence in Government | 0.11 | 0.03 | 4.34 | < 0.001 |
| Confidence in Civil Services | 0.14 | 0.02 | 6.33 | < 0.001 |
| Gender | 0.12 | 0.01 | 9.68 | < 0.001 |
| Age | 0.01 | < 0.001 | 16.99 | < 0.001 |
| Married | 0.06 | 0.01 | 5.55 | < 0.001 |
| College | 0.05 | 0.02 | 2.54 | 0.01 |
| Unemployed | -0.04 | 0.02 | -1.91 | 0.06 |
| Income Decile | -0.02 | < 0.01 | -5.86 | <0.001 |

*Note. R^2^ = 0.07. Country fixed effect coefficients are estimated but not reported.*

|  | Std. β | β | *SE* | *t* | *p* |
| --- | --- | --- | --- | --- | --- |
| Confidence in Government | .02 | 0.06 | 0.01 | 9.96 | < 0.001 |
| Confidence in Civil Services | .04 | 0.10 | < 0.01 | 19.79 | < 0.001 |

**Table C.** Linear regression using confidence in government and in civil services to predict tax compliance in OECD countries (*N* = 200,646).

*Note. R^2^ = 0.04. Country fixed effect coefficients are estimated but not reported.*

**Table D.** Linear regression using confidence in government and in civil services to predict tax compliance in non-OECD countries (*N* = 219,700).

|  | Std. β | β | *SE* | *t* | *p* |
| --- | --- | --- | --- | --- | --- |
| Confidence in Government | .03 | 0.05 | < 0.01 | 14.51 | < 0.001 |
| Confidence in Civil Services | .02 | 0.03 | < 0.01 | 8.37 | < 0.001 |

*Note. R^2^ = 0.09. Country fixed effect coefficients are estimated but not reported.*

**Table E.** Linear regression using confidence in government and in civil services to predict tax compliance in the United States (*N* = 9,938).

|  | Std. β | β | *SE* | *t* | *p* |
| --- | --- | --- | --- | --- | --- |
| Confidence in Government | < .01 | 0.01 | 0.03 | 0.46 | 0.64 |
| Confidence in Civil Services | .04 | 0.07 | 0.02 | 4.07 | < 0.001 |

*Note. R^2^ < 0.01*

**Table F.** Linear regression using confidence in government and in civil services to predict tax compliance in Canada (*N* = 6,711).

|  | Std. β | β | *SE* | *t* | *p* |
| --- | --- | --- | --- | --- | --- |
| Confidence in Government | .02 | 0.05 | 0.03 | 1.41 | 0.16 |
| Confidence in Civil Services | .06 | 0.12 | 0.02 | 4.87 | < 0.001 |

*Note. R^2^ < 0.01*

**Table G.** Mean confidence in government, confidence in civil services, and tax compliance in non–OECD countries, OECD countries, the United States, and Canada.

|  |  | Confidence in Gov’t | | | Confidence in Civil Services | | | Tax Compliance | | |
| --- | --- | --- | --- | --- | --- | --- | --- | --- | --- | --- |
|  | *N* | *Mean* | *SE* | *Mean* | | *SE* | *Mean* | | *SE* |  |
| Non–OECD | 219,700 | 0.15 | < .01 | 0.12 | | < .01 | 0.61 | | < .01 |  |
| OECD | 200,646 | 0.03 | < .01 | 0.05 | | < .01 | 0.58 | | < .01 |  |
| US | 9,938 | 0.03 | < .01 | 0.10 | | < .01 | 0.68 | | < .01 |  |
| Canada | 6,711 | 0.03 | < .01 | 0.06 | | < .01 | 0.65 | | < .01 |  |

**Table H.** Probit regression using both confidence in government and confidence in civil services to predict tax compliance across all countries (*N* = 420,346).

*Note. R^2^ = 0.05. Coefficients on country fixed effects are estimated but not reported.*

|  | β | *SE* | *t* | *p* |
| --- | --- | --- | --- | --- |
| Confidence in Government | 0.14 | 0.02 | 5.96 | < 0.001 |
| Confidence in Civil Services | 0.16 | 0.02 | 6.84 | < 0.001 |

**Table I.** Linear regression indicating how much countries differ from global average of tax compliance (*N* = 474,392).

| Country | β | *SE* | *t* | *p* |
| --- | --- | --- | --- | --- |
| Algeria | 0.09 | 0.01 | 7.32 | < 0.001 |
| Andorra | 0.11 | 0.02 | 6.59 | < 0.001 |
| Azerbaijan | 0.11 | 0.01 | 9.08 | < 0.001 |
| Argentina | 0.256 | 0.01 | 25.71 | < 0.001 |
| Australia | 0.12 | 0.01 | 12.09 | < 0.001 |
| Austria | 0.10 | 0.01 | 8.93 | < 0.001 |
| Bangladesh | 0.48 | 0.01 | 40.44 | < 0.001 |
| Armenia | 0.05 | 0.01 | 4.35 | < 0.001 |
| Belgium | –0.12 | 0.01 | –12.35 | < 0.001 |
| Bosnia | 0.25 | 0.01 | 20.82 | < 0.001 |
| Brazil | 0.06 | 0.01 | 5.75 | < 0.001 |
| Bulgaria | 0.13 | 0.01 | 12.24 | < 0.001 |
| Belarus | -0.13 | 0.01 | -13.35 | < 0.001 |
| Canada | 0.16 | 0.01 | 16.72 | < 0.001 |
| Chile | 0.20 | 0.01 | 19.65 | < 0.001 |
| China | 0.19 | 0.01 | 19.31 | < 0.001 |
| Taiwan | 0.15 | 0.01 | 12.88 | < 0.001 |
| Colombia | 0.25 | 0.01 | 27.06 | < 0.001 |
| Croatia | -0.01 | 0.01 | -0.54 | 0.591 |
| Cyprus | 0.24 | 0.01 | 21 | < 0.001 |
| Czech Republic | 0.07 | 0.01 | 6.86 | < 0.001 |
| Denmark | 0.14 | 0.01 | 13.61 | < 0.001 |
| Dominican Republic | 0.21 | 0.02 | 8.46 | < 0.001 |
| Ecuador | 0.13 | 0.02 | 8.12 | < 0.001 |
| El Salvador | 0.32 | 0.02 | 20.07 | < 0.001 |
| Ethiopia | 0.25 | 0.01 | 17.12 | < 0.001 |
| Estonia | 0.02 | 0.01 | 2.12 | 0.034 |
| Finland | 0.05 | 0.01 | 4.87 | < 0.001 |
| France | -0.01 | 0.01 | -1.45 | 0.147 |
| Georgia | 0.16 | 0.01 | 16.39 | < 0.001 |
| Palestine | -0.02 | 0.02 | -1.05 | 0.292 |
| Germany | 0.04 | 0.01 | 4.54 | < 0.001 |
| Ghana | 0.35 | 0.01 | 29.48 | < 0.001 |
| Greece | -0.02 | 0.01 | -2.04 | 0.041 |
| Guatemala | 0.05 | 0.02 | 2.82 | 0.005 |
| Hong Kong | 0.12 | 0.01 | 9.5 | < 0.001 |
| Hungary | 0.15 | 0.01 | 14.84 | < 0.001 |
| Iceland | 0.07 | 0.01 | 6.17 | < 0.001 |
| India | 0.18 | 0.01 | 19.23 | < 0.001 |
| Indonesia | 0.30 | 0.01 | 25.29 | < 0.001 |
| Iran | 0.27 | 0.01 | 26.21 | < 0.001 |
| Iraq | -0.17 | 0.02 | -10.38 | < 0.001 |
| Ireland | 0.01 | 0.01 | 1.26 | 0.209 |
| Italy | 0.10 | 0.01 | 10.73 | < 0.001 |
| Japan | 0.34 | 0.01 | 35.45 | < 0.001 |
| Kazakhstan | 0.05 | 0.01 | 3.56 | < 0.001 |
| Jordan | 0.33 | 0.01 | 28.87 | < 0.001 |
| South Korea | 0.26 | 0.01 | 26.49 | < 0.001 |
| Kuwait | 0.06 | 0.02 | 3.54 | < 0.001 |
| Kyrgyzstan | 0.06 | 0.01 | 4.91 | < 0.001 |
| Lebanon | -0.06 | 0.02 | -3.98 | < 0.001 |
| Latvia | -0.03 | 0.01 | -2.48 | 0.013 |
| Libya | 0.32 | 0.01 | 24.44 | < 0.001 |
| Lithuania | -0.07 | 0.01 | -6.8 | < 0.001 |
| Luxembourg | -0.02 | 0.01 | -1.28 | 0.200 |
| Malaysia | -0.09 | 0.01 | -7.01 | < 0.001 |
| Mali | 0.05 | 0.01 | 3.42 | 0.001 |
| Malta | 0.31 | 0.01 | 26.86 | < 0.001 |
| Mexico | 0.07 | < 0.01 | 7.07 | < 0.001 |
| Moldova | -0.09 | 0.01 | -8.1 | < 0.001 |
| Montenegro | 0.10 | 0.01 | 8.58 | < 0.001 |
| Morocco | 0.36 | 0.01 | 31.67 | < 0.001 |
| Netherlands | 0.04 | 0.01 | 3.82 | < 0.001 |
| New Zealand | 0.12 | 0.01 | 10.32 | < 0.001 |
| Nigeria | 0.10 | 0.01 | 9.86 | < 0.001 |
| Norway | -0.04 | 0.01 | -3.45 | 0.001 |
| Pakistan | 0.32 | 0.01 | 27.19 | < 0.001 |
| Peru | 0.12 | 0.01 | 11.22 | < 0.001 |
| Philippines | -0.09 | 0.01 | -8.27 | < 0.001 |
| Poland | 0.03 | 0.01 | 3.02 | 0.003 |
| Portugal | -0.01 | 0.01 | -0.86 | 0.388 |
| Puerto Rico | 0.26 | 0.01 | 19.28 | < 0.001 |
| Romania | 0.13 | 0.01 | 13.74 | < 0.001 |
| Russia | -0.03 | < 0.01 | -2.82 | 0.005 |
| Rwanda | -0.09 | 0.01 | -8.05 | < 0.001 |
| Serbia | 0.15 | 0.01 | 14.06 | < 0.001 |
| Singapore | 0.08 | 0.01 | 7.16 | < 0.001 |
| Slovakia | -0.01 | 0.01 | -1.18 | 0.239 |
| Vietnam | 0.32 | 0.01 | 25.48 | < 0.001 |
| Slovenia | 0.12 | 0.01 | 12.49 | < 0.001 |
| South Africa | 0.05 | < 0.01 | 5.57 | < 0.001 |
| Zimbabwe | 0.16 | 0.01 | 12.89 | < 0.001 |
| Spain | 0.07 | < 0.01 | 8.26 | < 0.001 |
| Sweden | 0.06 | 0.01 | 6.5 | < 0.001 |
| Switzerland | 0.09 | 0.01 | 8.59 | < 0.001 |
| Thailand | -0.02 | 0.01 | -1.89 | 0.058 |
| Trinidad/Tobago | 0.25 | 0.01 | 18.56 | < 0.001 |
| Tunisia | 0.16 | 0.02 | 10.13 | < 0.001 |
| Turkey | 0.37 | 0.01 | 38.27 | < 0.001 |
| Uganda | 0.06 | 0.02 | 3.38 | 0.001 |
| Ukraine | -0.04 | 0.01 | -4.54 | < 0.001 |
| Macedonia | 0.22 | 0.01 | 18.98 | < 0.001 |
| Egypt | 0.25 | 0.01 | 25.6 | < 0.001 |
| Great Britain | 0.09 | 0.01 | 8.69 | < 0.001 |
| Tanzania | 0.36 | 0.02 | 22.34 | < 0.001 |
| United States | 0.19 | 0.01 | 19.96 | < 0.001 |
| Burkina Faso | 0.06 | 0.02 | 3.81 | < 0.001 |
| Uruguay | 0.26 | 0.01 | 21.89 | < 0.001 |
| Uzbekistan | 0.10 | 0.01 | 6.67 | < 0.001 |
| Venezuela | 0.21 | 0.01 | 16.59 | < 0.001 |
| Yemen | 0.18 | 0.02 | 10.15 | < 0.001 |
| Serbia/  Montenegro | -0.17 | 0.02 | -10.68 | < 0.001 |
| Zambia | -0.15 | 0.01 | -10.16 | < 0.001 |
| North Ireland | 0.10 | 0.01 | 7.8 | < 0.001 |
| Bosnian Federation | 0.14 | 0.02 | 7.47 | < 0.001 |
| Kosovo | 0.37 | 0.01 | 25.57 | < 0.001 |

*Note. R^2^ = 0.07*
